# The Dustbin in "Progressive" Putney

**Published:** 1904-12-31

**Authors:** 


### THE DUSTBIN IN "PROGRESSIVE" PUTNEY.

From a Correspondent.

Recently the Wandsworth Borough Council announced its intention of making a daily collection of house refuse instead of leaving it to accumulate for a week in the time-honoured fashion. Simultaneously there came a by-election for the Putney Ward of the borough. Putney has always been Conservative in its instincts, and the chances are that it would have returned a member of that party if the "Progressive" candidate (save the mark!) had not—to show his progressiveness, no doubt—placarded and post-carded the neighbourhood with his promise to oppose strongly "the absurd proposals for the daily removal of house refuse." The pledge thus volunteered made such a profound impression on the electorate that the Conservative candidate sent out a guarded promise not to support the new method. But as he was an architect, and therefore had a knowledge of sanitary matters, he could not go as far as his rival and condemn the proposal as absurd. Putney was thrilled to its depths. The female electorate, to a woman, vowed to support the protector of the dustbin. Servants, hearing that under the new regulations the receptacle for dust would need to be placed on the street by some member of the household, gave notice that they would not touch it. One prominent householder declared that he would leave the neighbourhood sooner than be deprived of the privilege of accumulating his ashes for a week; another vowed he would change his politics rather than lose his dustin. An evening paper plunged into the fray and dwelt upon the unpleasantness to the men of Putney if they had to pass by a row of dustbins on their way to the train in the morning. The writer apparently did not realise either that refuse, if collected daily, is not so offensive as if allowed to decompose for a week, or that the nostrils of women and children might suffer equally if the refuse were allowed to remain in proximity to the house. Perhaps the borough council was ill-advised to introduce the change in the method of dust-collection in the winter, when the offensiveness of the accumulation of refuse is less marked than in summer, both because the weather is colder and because much of the most troublesome kind can be rendered innocuous by burning in the kitchen fire. In the summer, when many people use gas cooking-stoves and the sun is strong, every woman is conscious of the disagreeableness of her neighbour's dustbin if not of her own, and the change might have been received even gratefully. But fate, wishing to encourage the Putney Liberals, arranged the alteration to take place just now, and, consequently, that party to-day is rejoicing in the return of a "Progressive" councillor as the apostle of insanitary conservatism!

